# Growth of emergent simple pseudo-binary ferroelectrics and their potential in neuromorphic computing devices

**DOI:** 10.1039/d4mh00153b

**Published:** 2024-02-27

**Authors:** Ampattu R. Jayakrishnan, Ji S. Kim, Markus Hellenbrand, Luís S. Marques, Judith L. MacManus-Driscoll, José P. B. Silva

**Affiliations:** a Physics Center of Minho and Porto Universities (CF-UM-UP), University of Minho, Campus de Gualtar 4710-057 Braga Portugal josesilva@fisica.uminho.pt; b Laboratory of Physics for Materials and Emergent Technologies, LapMET, University of Minho 4710-057 Braga Portugal; c Dept. of Materials Science and Metallurgy, University of Cambridge 27 Charles Babbage Rd. Cambridge CB3 OFS UK jld35@cam.ac.uk

## Abstract

Ferroelectric memory devices such as ferroelectric memristors, ferroelectric tunnel junctions, and field-effect transistors are considered among the most promising candidates for neuromorphic computing devices. The promise arises from their defect-independent switching mechanism, low energy consumption and high power efficiency, and important properties being aimed for are reliable switching at high speed, excellent endurance, retention, and compatibility with complementary metal–oxide–semiconductor (CMOS) technology. Binary or doped binary materials have emerged over conventional complex-composition ferroelectrics as an optimum solution, particularly in terms of CMOS compatibility. The current state-of-the-art route to achieving superlative ferroelectric performance of binary oxides is to induce ferroelectricity at the nanoscale, *e.g.*, in ultra-thin films of doped HfO_2_, ZrO_2_, Zn_1−*x*_Mg_*x*_O, A_l−*x*_Sc_*x*_N, and Bi_1−*x*_Sm_*x*_O_3_. This short review article focuses on the materials science of emerging new ferroelectric materials, including their different properties such as remanent polarization, coercive field, endurance, *etc.* The potential of these materials is discussed for neuromorphic applications.

## Abstract 2

Ferroelectric memory devices such as ferroelectric memristors, ferroelectric tunnel junctions, and field-effect transistors are considered among the most promising candidates for neuromorphic computing devices.

## Graphical abstract

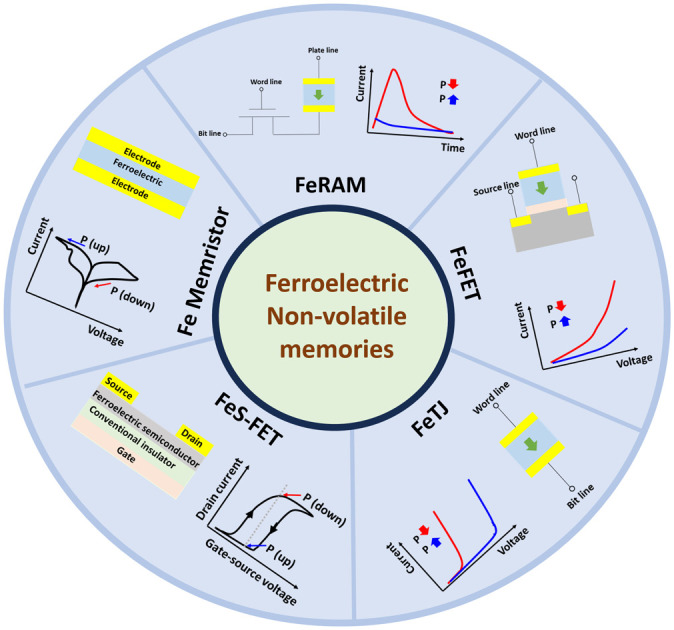

**Wider impact**

Ferroelectric memory devices are considered among the most promising candidates for neuromorphic computing. The promise arises from their defect-independent switching mechanism, low energy consumption and high power efficiency, and important properties being aimed for are reliable switching at high speed, excellent endurance, retention, and compatibility with complementary metal–oxide–semiconductor (CMOS) technology, making them one of the most suitable candidates for neuroinspired computing applications. In this short review article we highlight the materials science of emerging new ferroelectric materials, such as doped HfO_2_, ZrO_2_, Zn_1−*x*_Mg_x_O, A_l−*x*_Sc_*x*_N, and Bi_1−*x*_Sm_*x*_O_3_, including their different properties such as remanent polarisation, coercive field, endurance, *etc.* The potential of these materials is emphasized for neuromorphic applications, particularly in terms of CMOS compatibility. We also discuss possible routes for materials optimization to achieve superior neuromorphic performance.

## 1. Neuromorphic computing and the promise of ferroelectric devices

**Fig. 1:**
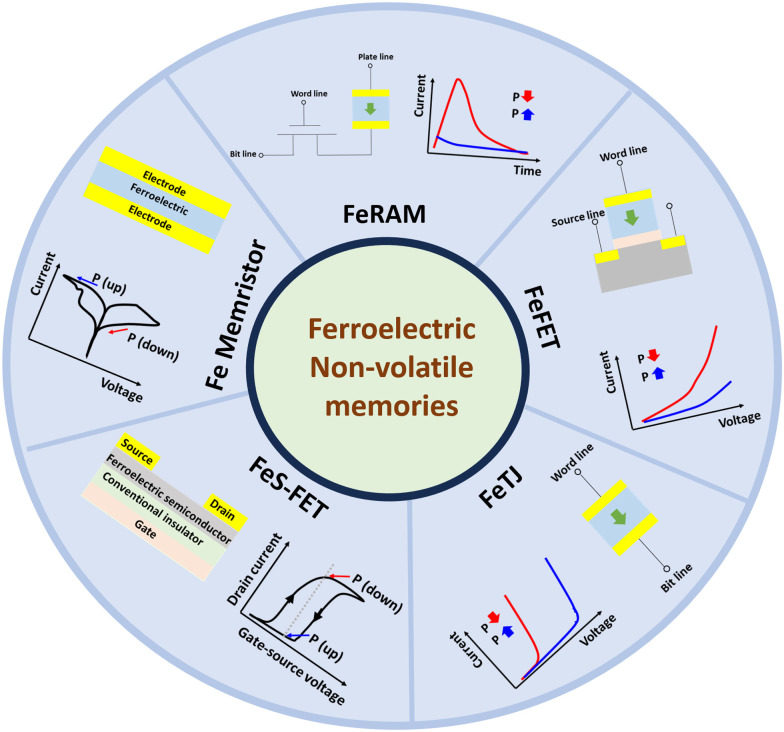
Different types of ferroelectric non-volatile memories that are applicable to neuromorphic computing and their corresponding current responses.

Owing to the exponential increase in the quantity of information to be stored and processed,^1–4^ there is a need for lower energy, faster and more cost-effective computing. To achieve this, new computing paradigms are highly sought after. The conventional von Neumann architecture, which is currently required to perform processing of large quantities of data, leads to low energy efficiency attributable to the separation of processing and memory units, which leads to a high energy cost when moving data back and forth between the two.^3,4^ The imminent end of Moore's Law will also limit computing capacity. Although in its early stage, neuromorphic, ‘brain-like’, computing has received significant attention as a possible alternative for undertaking data-intensive cognitive tasks quickly and efficiently. In neuromorphic computing systems, the two key structures are artificial neural network (ANN) and spiking neural network (SNN) systems to process and store information in the same place, thus eliminating the energy-intensive data shuttling between the two.^2–4^ An analog synaptic device can be used to emulate the synaptic functionality in ANNs or SNNs for realizing cognitive tasks such as parallel computation and adaptive learning.^3,4^ The analog synaptic device is crucial in neuromorphic computing as a device with an effectively controllable conductance, which can be successfully arranged in a crossbar array to execute ‘neural’ tasks such as matrix vector multiplication. A multi-level non-volatile memory element can be used as an analog synaptic device and has the potential to implement potentiation (ability to gradually increase the conductance upon voltage pulses) and depression (ability to gradually decrease the conductance upon voltage pulses) for short-term and long-term plasticity. Non-volatile memory (NVM) devices can be based on resistive switching, phase change, and magnetic and ferroelectric materials, amongst others, and can imitate synapses by using just a single device or a few devices. Among them, ferroelectric-material-based memories such as ferroelectric random access memory (FeRAM), ferroelectric field-effect transistors (FeFETs, FETs with ferroelectric gate dielectrics), ferroelectric semiconductor field-effect transistors (FeS-FETs, FETs with ferroelectric semiconductor channels), ferroelectric tunnel junctions (FeTJs), and ferroelectric memristors (Fe memristors) are the most promising contenders owing to their inherent benefits such as a defect-independent switching mechanism, low energy consumption, and good power efficiency for neuromorphic computing systems.^4–6^Fig. 1 provides a schematic to illustrate the structures and functionalities of these devices.

## 2. Evolution of ferroelectric materials for non-volatile memory devices

**Fig. 2:**
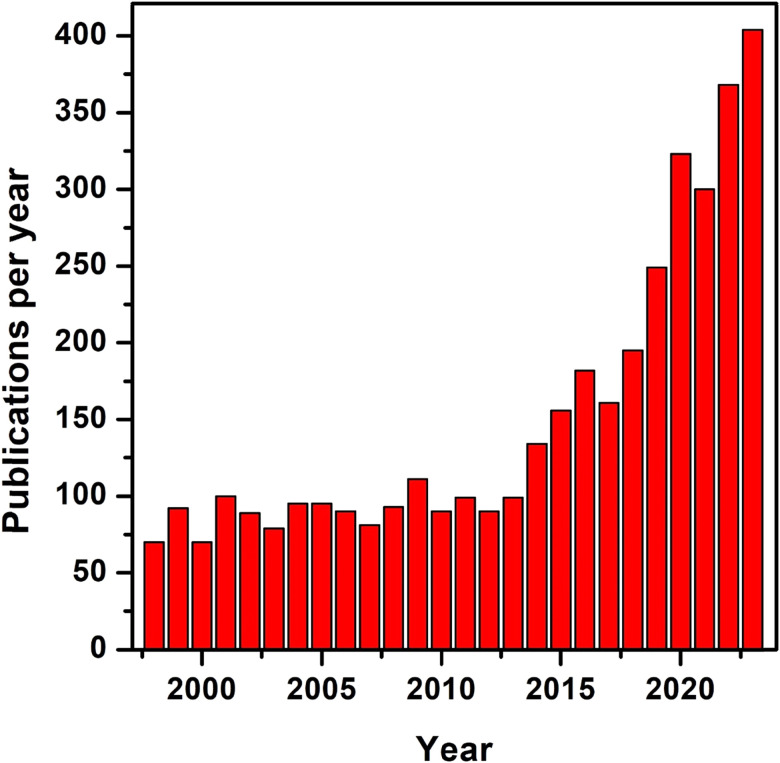
Publications per year based on ferroelectric memories from the year 1998 up to 2023 [Analyze Results (webofscience.com)].

In the electronics area, recent applications include ferroelectric non-volatile memory devices, which can be considered as the basic building blocks for neuromorphic computing devices.^4,7,8^ Ferroelectric materials possess the ability to switch, upon appropriate voltage application, the polarization direction of their remanent polarization (*P*_r_), which is stable at zero electric field. This offers features like fast read/write speed, low power consumption, and high reliability compared with conventional floating-gate electrically erasable programmable read-only memory (EEPROM) and flash EEPROM devices, which require long read/write times and high voltages.^8^ Furthermore, ferroelectric materials can offer unique gradual domain switching characteristics, which provide the aforementioned multi-level conductance/resistance functionality. As a result, there is an increased interest in ferroelectric materials for non-volatile memory and neuromorphic applications. Fig. 2 shows the evolution of the number of publications per year since 1998, using the keywords ‘ferroelectric materials and memory applications’ in a Web of Science search. Up to 2013, the number of publications remained almost constant and then increased exponentially. This is linked to the discovery of new ferroelectric materials, as discussed below.

Perovskite-based ferroelectric materials such as lead zirconate titanate (PZT) and strontium bismuth tantalate (SBT) are promising materials for achieving the performance required for memory applications such as logic-in memory (LiM) and non-volatile logic devices.^9–17^ For instance, different companies (such as Cypress Semiconductor, Texas Instruments, and Fujitsu) installed a PZT-based FeRAM in applications, including wearable medical gadgets, smart cards, energy meters, airplane black boxes, radio frequency tags, and code storage in microcontrollers.^11^ SBT-based FeFETs with a huge memory window and almost infinite cycling endurance (>10^12^) were demonstrated by Sakai *et al.*,^12^ which later exhibited adequate operation in non-volatile logic (NVL) circuits^12^ and 64 kbit NAND memory arrays.^13^ However, while recent investigations concentrated on the continued physical scaling-down of the metal–oxide–semiconductor field-effect transistor (MOSFET),^13,15^ the integration of perovskite oxides with front-end semiconductor manufacturing processes faced difficulties, particularly those related to perovskite etching, high crystallization temperature, hydrogen sensitivity, thickness, and cell size scaling beyond the 130 nm technology node.^9,11^ For instance, PZT experiences scalability problems,^9,11^ because it often loses its polarization in thin films at the nanoscale due to large depolarization fields. Increasingly, 3D integration is required for high-density memory technology with constantly decreasing dimensions, which exacerbates the scaling difficulties for perovskite ferroelectric films at the industrial level.^11^ Furthermore, PZT contains lead, which makes it toxic with serious health implications.

**Fig. 3:**
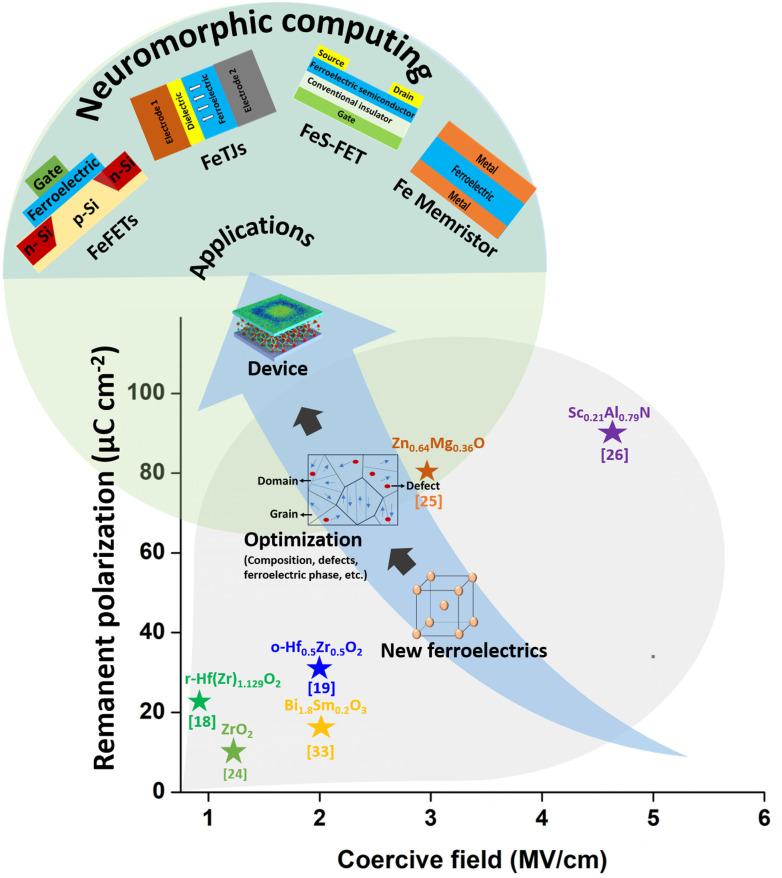
New ferroelectric materials and their possible applications. o- and r- indicate orthorhombic and rhombohedral phases, respectively.

Considering the aforementioned challenges of perovskites, the discovery of ferroelectricity in Si-doped hafnium oxide (Si:HfO_2_) thin films has provided a promising alternative to resolve these problems.^15,16^ Following this exciting discovery, ferroelectricity in the nano-regime in Si:HfO_2_ has attracted significant attention from the ferroelectrics community due to its compatibility with the complementary metal–oxide–semiconductor (CMOS) technology. Inducing ferroelectricity at the nanoscale is the current trend in non-volatile memories to realize applications such as high-density memory, storage class memory, neuromorphic computing, hardware security, *etc.*^17^ Recent progress in this field has introduced new ferroelectric materials besides Si:HfO_2_ with promising ferroelectric polarization and superior scalability to the nanoscale regime that are compatible with CMOS for the next-generation ferroelectric memories as shown in Fig. 3.^18–34^Fig. 3 also provides some keywords for the enhancement of *P*_r_ with moderate coercive field (*E*_c_) values (1–2 MV cm^−1^), which can induce large memory windows in non-volatile memory devices such as FeFETs for non-destructive readout operations.^1^ For FeFETs, however, a moderate *P*_r_ is already ideal, as too high of a *P*_r_ value can deteriorate the device reliability due to a high depolarization field and a high concentration of trapped charges.^1^Fig. 3 clearly provides a picture that orthorhombic (o-) Hf_0.5_Zr_0.5_O_2_ is at an advanced stage compared with other ferroelectric materials in terms of achieving good *P*_r_, low *E*_c_, and excellent CMOS compatibility. Table 1 complements Fig. 3, showing further details such as preparation methods, electrode materials, and film thicknesses, which were used in the ferroelectric-based devices, together with other relevant key ferroelectric performance indicators such as required wake-up cycles (the number of switching cycles required to achieve the maximum *P*_r_), endurance, Curie temperature (*T*_c_), *etc.* With this as the background, this short review focuses on the breakthroughs in the newly introduced ferroelectric materials from the viewpoint of their ferroelectric properties and how they are beneficial for neuromorphic applications. Finally, we conclude the review article with a perspective on this burgeoning area.

**Table 1:** Key reports on (pseudo-)binary ferroelectric materials and their properties

Material and thickness	Method	*P*_r_ (μC cm^−2^)	*E*_c_ (MV cm^−1^)	*T*_c_ (K)	Wake-up (cycle)	Endurance (cycles)	Top/bottom electrodes	Substrate	Ref.
Hf(Zr)_1.129_O_2_ (12 nm)	Sputtering	22	0.65	—	None	10^12^	TiN (top and bottom)	Si	18
Hf_0.5_Zr_0.5_O_2_ (20 nm)	ALD	∼1.07	∼19	—	Needed	10^4^	Ni/Pt	SiO_2_/Si	19
Hf_0.5_Zr_0.5_O_2_ (10 nm)	ALD	∼30	∼2	—	None	10^4^	W (top and bottom)	SiO_2_/Si	19
Hf_0.5_Zr_0.5_O_2_ (11 nm)	Sputtering	∼1	∼10	—	Needed	10^4^	TiN/Pt	SiO_2_/Si	20
Hf_0.5_Zr_0.5_O_2_ (9 nm)	ALD	∼1	∼21.7	—	Needed	10^5^	TiN/Pt	SiO_2_/Si	21
Hf_0.5_Zr_0.5_O_2_ (5 nm)	PLD	∼5	∼34	—	None	—	La_0.7_Sr_0.3_MnO_3_ (top and bottom)	SrTiO_3_	22
ZrO_2_ (30 nm)	CVM	8.5	—	—	—	10^3^	TiN/Pt	Si (100)	23
ZrO_2_ (8 nm)	IBSD	9.3	1.2	—	None	10^6^	Au (top)	Nb:SrTiO_3_	24
Zn_0.64_Mg_0.36_O (300 nm)	Sputtering	80	3	—	20	10^3^	Pt (top and bottom)	SiO_2_/Si	25
Al_0.79_Sc_0.21_N (100 nm)	MBE	90	4.6	—	—	10^5^	Au/n-type GaN	Si	26
Al_0.7_Sc_0.30_N (45 nm)	Sputtering	100	>5	—	—	—	TiN (top and bottom)	SiO_2_/Si	27
Al_0.64_Sc_0.36_N (30 nm)	Sputtering	100	>6	—	—	<10^5^	TiN (top and bottom)	SiO_2_/Si	28
Al_0.64_Sc_0.36_N (16 nm)	Sputtering	30	6.5	—	—	—	Pt (top and bottom)	SiO_2_/Si	29
Sc_0.30_Al_0.7_N (5 nm)	MBE	23	>6	—	—	—	Mo (top and bottom)	Mo	30
Al_0.72_Sc_0.28_N (5 nm)	PVD	>100	9.2	—	—	—	Al (top and bottom)	Sapphire	31
Al_0.64_Sc_0.36_N (100 nm)	Sputtering	80	2	—	—	10^4^	Ti–Au/Pt	Si	32
Bi_1.8_Sm_0.2_O_3_ (1 nm)	Sol–gel	17	∼2	473	—	10^8^	Au (top)	Nb:SrTiO_3_	33
Bi_1.8_Sm_0.2_O_3_ (4.6 nm)	CSM	50	0.75	—	—	10^9^	Au/Cr (top)	Nb:SrTiO_3_	34

## 3. Novel ferroelectric materials for neuromorphics

### 3.1 HfO_2_ and ZrO_2_-based films

Since the discovery of ferroelectricity in Si-doped hafnium oxide in 2011, doped hafnium oxide has been receiving significant attention.^35^ The discovery of CMOS-compatible and scalable ferroelectric materials has excited the ferroelectrics community.^35,36^ Following this, there have been extensive investigations into its fundamentals and applications. Hafnium oxide typically adopts a stable monoclinic phase (*P*2_1_/*c*) at room temperature, a tetragonal (*P*4_2_/*mnc*) structure at >2100 K, and a cubic (*Fm*3̄*m*) structure at >2800 K.^35,36^ These phases are non-polar, *i.e.*, non-ferroelectric. So far, there have been a tremendous number of studies, experimental and computational, investigating the stabilization of meta-stable polar phases. Ferroelectricity in hafnium- and zirconium-based oxides arises from meta-stable, polar orthorhombic (o-) or rhombohedral (r-) phases with space groups *Pca*2_1_ or *R*3*m*.^22,37^ In addition, several factors including doping, deposition method, post-deposition treatment, interface chemistry, *etc.* have shown to enhance the ferroelectric performance of these materials.^38–40^ In terms of doping, numerous dopants have been investigated such as Si, La, Gd, Y, Sr, Al, and Zr.^38–47^ Out of all the dopants in hafnium oxide, zirconium shows the most promise owing to the robust ferroelectric properties upon introducing it. Wei *et al.* reported wake-up-free ferroelectric switching in ultra-thin Hf_0.5_Zr_0.5_O_2_ films (thickness of 5 nm) by stabilizing the ferroelectric r-phase using a compressive epitaxial strain engineering strategy.^22^ Recently, the same group reported that the polarization switching in r-phase Hf_0.5_Zr_0.5_O_2_ films (thickness of 6 nm) might be ascribed to reversible oxygen vacancy migration.^45^ In another work, Stylianidis *et al.* reported ferroelectricity in 5-nm-thick Hf_0.5_Zr_0.5_O_2_ films also attributable to the polarization switching driven by oxygen vacancy migration.^46^ Yun *et al.*, on the other hand, confirmed polarization switching in yttrium-doped HfO_2_ with an o-phase with a small rhombohedral distortion attributable to “true” ferroelectricity, *i.e.*, the reversible switching of the material's unit cell polarization.^47^ The electrical switchability of the yttrium-doped HfO_2_ films studied using piezo-response force microscopy revealed features of “intrinsic” ferroelectricity not obscured by extrinsic factors like charge injection. Overall, the phenomenon of ferroelectric switching in hafnia-based films is still under debate regarding the origin of the observed polarization switching.^45,46^ The minimum film thickness that demonstrated ferroelectricity in Zr-doped HfO_2_ (Hf_1−*x*_Zr_*x*_O_2_) with *x* = 0.2 was 1 nm, which was grown by low-temperature atomic layer deposition (ALD) on a silicon substrate, followed by rapid thermal annealing.^48^ Furthermore, the Zr-doped composition Hf_1−*x*_Zr_*x*_O_2_, with *x* = 0.5, is the most promising one for its excellent ferroelectric properties attributable to the stable ferroelectric orthorhombic phase (*Pbc*2_1_) due to the ferroelectric phase transition in the HfO_2_–ZrO_2_ solid solution.^38^

There have been many attempts to deposit ferroelectric Hf_0.5_Zr_0.5_O_2_ (HZO) using various deposition techniques such as ALD, pulsed laser deposition (PLD), metal–organic chemical vapor deposition (MOCVD), or sputtering.^49–56^ In the early stages of research, most HZO films were deposited using ALD on top of Si, SiO_2_ or TiN, followed by a rapid thermal annealing (RTA) step.^39–41^ Generally, an *E*_c_ value of ∼1 MV cm^−1^ and a remanent polarization *P*_r_ value of >15 μC cm^−2^ were achieved.^45,47^ ALD-grown HZO films tend to demonstrate wake-up behavior, which means that they require a number of applied voltage cycles prior to the actual demonstration of ferroelectric behavior or before reaching their maximum *P*_r_ value. There have been multiple attempts to mitigate this wake-up effect and some reports demonstrated wake-up-free ALD-grown HZO through capping electrode engineering, mechanical stress, and plasma treatment.^10^ Furthermore, epitaxial HZO has been investigated, mainly using PLD. While PLD is currently not CMOS-compatible like ALD, it enables understanding of fundamental properties about the origin of ferroelectricity in HZO with highly crystallized single-phase films. HZO films have been grown on SrTiO_3_ (STO) (001) with the La_0.67_Sr_0.33_MnO_3_ (LSMO) buffer layer^55–58^ and they typically demonstrate o-(111) oriented films. However, in 2018, Y. Wei *et al.* reported r-(111) ferroelectric films for the first time.^28^ The factors determining the formation of a specific ferroelectric phase remain unclear. Recent studies conducted by S. Estandia *et al.*, 2021,^57^ and S. Shi *et al.*, 2023,^58^ have highlighted the significant role of the bottom electrode in achieving the ferroelectric o-phase. Their findings indicate that MnO termination of LSMO is necessary for the formation of the o-phase. Epitaxial o-HZO films tend to have a high *E*_c_ of >3 MV cm^−1^ (depending on their thickness) and *P*_r_ > 30 μC cm^−2^. It is unclear at present which termination or other structural aspects of LSMO are needed to achieve the r-phase. In addition, recently, magnetron-sputtered r-Hf(Zr_1.129_)O_2_ films have shown a very good *P*_r_ of 22 μC cm^−2^, an ultra-low *E*_c_ of 0.65 MV cm^−1^, and stable performance up to ∼10^12^ cycles, which seem to solve the problem of high *E*_c_ in o-phase HfO_2_-based films. The demonstrated stabilization of the r-phase and its associated ferroelectric properties are attributed to intercalated atoms that expand the lattice, thereby increasing both in-plane and out-of-plane stresses, thus creating a more highly polarised structure, which is easier to switch due to its lower kinetic energy barrier between the two spontaneous polarization states.^18^

Rapid development of nanoscale HfO_2_-based materials with excellent ferroelectric properties and available multi-level conductance states, along with their physical dimensions and compatibility with CMOS technology, has inspired the adoption of these materials into synaptic devices with low footprints and high energy efficiency.^49–56^ The key parameters that determine the ideal requirement for a synaptic device are high *P*_r_, moderate *E*_c_, large on/off ratio, long cycling endurance, prolonged retention, and well-spaced conductance levels.^4^ For instance, ferroelectric materials can achieve multi-level *P*_r_ states by varying the applied electric switching field. Not only that, these materials maintain each state even after removal of the electric field. This multi-level *P*_r_ can be used to modulate the conductance of the synaptic device.^59,60^ The low *E*_c_ can extend the endurance lifetime and result in low-voltage switching for non-volatile memories.^61^ A large on/off ratio potentially increases the number of multi-level states by providing a large memory window.^4,62^ Moreover, the endurance and retention of the ferroelectric materials allow the memory device to store the data over a long time.^62^ Therefore, we can say that ferroelectric-based non-volatile memory devices are promising for synaptic elements in neuromorphic computing.

Recently, researchers have reported analog synaptic transistors based on HfO_2_- and ZrO_2_-based ferroelectric materials, which employ a variety of channel materials, including WO_*x*_, indium gallium zinc oxide (IGZO), indium zinc tin oxide (IZTO), poly-GeSn, and WS_2_.^63–69^ HfO_2_- and ZrO_2_-based ferroelectric materials are attractive due to their ferroelectricity in ultra-thin films (from ∼1 up to 10 nm) with improved reliability, low programming voltage and CMOS compatibility.^63–66^ For instance, Halter *et al.* studied an Hf_0.57_Zr_0.43_O_2_ thin-film-based (thickness ∼9.6 nm) FeFET with WO_*x*_ as the channel material for synaptic elements.^63^ This work showcased an Hf_0.57_Zr_0.43_O_2_/WO_*x*_ stack as an FeFET with good voltage control and strong linearity and symmetry in potentiation and depression at different voltage amplitudes with identical pulse trains, which facilitate facile programming of learning algorithms. The FeFET has a short programming time of 40 ns and a low write energy (2.1 × 10^−17^ J μm^−2^). Furthermore, it offers multi-level programming spanning more than 4 bit depth and a stable retention over 1500 s. Moreover, the device is compatible with a back end of line (BEOL) integration into standard CMOS processes. Kim *et al.* reported Al-doped HfO_2_ with improved switching speed (∼107 mV per decade) and a good on/off ratio (1 × 10^3^), demonstrating its use as a potential synaptic transistor.^65^ By varying the amplitude and number of input pulses, this work was able to achieve potentiation/depression weight updates with minimal cycle-to-cycle variability and good linearity. With the performance data of this FeFET, a simulated pattern recognition accuracy of almost 90% was achieved for the Modified National Institute of Standard and Technology (MNIST) handwritten data set under optimal potentiation/depression conditions. Furthermore, Al-doped HfO_2_ suppresses electrically active charge traps typically arising from the direct deposition of HfO_2_ on Si and favors analog conduction modulation at low operating voltages.

**Fig. 4:**
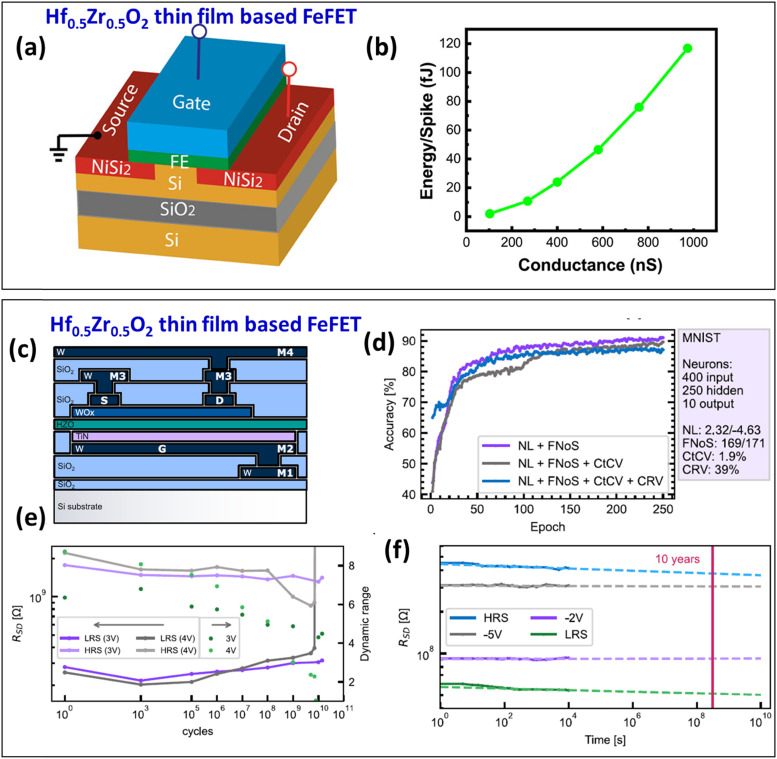
(a) Schematic diagram of an Hf_0.5_Zr_0.5_O_2_ thin-film-based 3-terminal FeFET. (b) Calculated energy/spike consumption at different voltage pulse amplitudes.^68^ Copyright (2020) by American Chemical Society. (c) Illustration of an FeFET device based on an Hf_0.5_Zr_0.5_O_2_ thin film with source (S), drain (D), gate (G), WO_*x*_ channels, ferroelectric gate dielectric, access metal lines (M1, M2), plus S access metal line (M3) and D access metal line (M4). (d) MNIST classification performance of the FeFET with different degrees of non-idealities included: non-linearity factors and finite number of steps (purple), added cycle to cycle variation (grey), and added conductance range variation (blue). (e) Endurance measurement of the FeFET at 100 kHz. (f) Retention measurements at room temperature for the four programmed states^69^ with extrapolation to 10 years.

Bégon-Lours *et al.* developed a 3.5-nm-thick Hf_0.5_Zr_0.5_O_2_ thin film transistor with an on/off ratio of 7 and a small device-to-device variability (<5%) suitable for neural network inference.^67^ Furthermore, the device operates in the ohmic regime for the read-out, which is good for analog vector-matrix multiplication. The conduction modulation in the transistor may have originated from the motion of trapped hydrogen in the lattice and/or oxygen vacancies that form during ALD fabrication. These defects modify the conduction band energy profile and transport properties upon polarization switching. As a synaptic element, this transistor demonstrated a training accuracy of 92% after 36 epochs on the MNIST data set. Xi *et al.* experimentally demonstrated a CMOS-compatible FeFET using an Hf_0.5_Zr_0.5_O_2_ ferroelectric layer (thickness ∼10 nm, Fig. 4(a)) with a very high on/off ratio (3 × 10^4^) and low write energy consumption (∼2 fJ) at low voltages (0.5 V) (Fig. 4(b)).^68^ This synaptic device was based on Hf_0.5_Zr_0.5_O_2_/NiSi_2_/Si and used partial polarization switching to modulate the Schottky barrier of the NiSi_2_/Si contacts for analog conduction modulation with symmetric potentiation/depression characteristics and high retention.

Very recently, Halter *et al.* reported an Hf_0.5_Zr_0.5_O_2_ thin film FeFET (thickness ∼10 nm) (Fig. 4(c)) with good *P*_r_ (∼14 μC cm^−2^) and a very low write energy (1.2 fJ) at higher voltages (6 V).^69^ In terms of neuromorphic behavior, using the NeuroSim framework, a good MNIST classification accuracy of 88% was achieved (Fig. 4(d)) due to fine-grained, quasi-continuous monotonic resistance changes with more than 200 steps between the low and high resistance states and a low cycle-to-cycle variability. Furthermore, a very minimal dynamic range loss with a very high endurance (>10^10^ cycles) and an outstanding retention (>10 years) was achieved (Fig. 4(e) and (f)). As a result, the FeFET is promising for neural network inference and cognitive computing.

In a different study, Mikheev *et al.* fabricated a p-type Si/Hf_0.5_Zr_0.5_O_2_/TiN-based second-order memristor using a 4-nm-thin ferroelectric Hf_0.5_Zr_0.5_O_2_ ultra-thin film to investigate the synaptic functions for neuromorphic computing.^70^ In first-order memristors, the conductance is defined entirely by external stimuli and thus to emulate the temporal synapse response, an accurate overlapping of pre- and post-synaptic spikes is needed, which is not the case in biological synapses. In second-order memristors, the conductance is controlled by both the external stimuli and their instant internal state. These second-order memristors do not require particular spike shapes and their overlapping and thus allow a straightforward natural emulation of the frequency response. Mikheev *et al.* demonstrated conductivity (synaptic weight) modulation with *R*_OFF_/*R*_ON_ ∼ 8 *via* the gradual switching of polarization in ferroelectric domains of polycrystalline 4-nm-thin ferroelectric Hf_0.5_Zr_0.5_O_2_ films. The built-in electric field and charge trapping/de-trapping at the defect states at the Si interface contribute further to the temporal behavior, expressing modulation similar to synapses in biological systems. The work of Mikheev *et al.* also experimentally demonstrated synaptic functions such as short-term plasticity, paired-pulse facilitation (PPF), paired-pulse depression (PPD), and spike-rate-dependent plasticity (SRDP), which are similar to biological synaptic systems. Müller *et al.* developed another promising approach using an Fe memristor for the prospects of neuromorphic applications. A simple metal–ferroelectric–metal (MFM) structure utilizing an ultra-thin Hf_0.93_Y_0.07_O_2_ film of thickness 4.5 nm displayed a very good on/off ratio (540) and a large range of accessible resistance states for future NVM.^66^ This work introduces polarization-modulated charge transport from Schottky-barrier-limited to Ohmic conduction, reporting a high on/off ratio for ultrathin epitaxial Hf_0.93_Y_0.07_O_2_ films. The impact of the polarization reversal on the charge transport across a MFM device, utilizing a partially depleted insulating ferroelectric barrier, is demonstrated, which is conceptually different from those in FeTJs, where a fully depleted insulating ferroelectric barrier is used.

From these examples, it can be concluded that these HfO_2_- and ZrO_2_-based materials are all viable options for large-scale integration into neuromorphic hardware. However, one of the critical outstanding challenges with HfO_2_ and ZrO_2_ FeFETs are wake-up effects, about which no information has been provided in any of the reviewed examples.

**Fig. 5:**
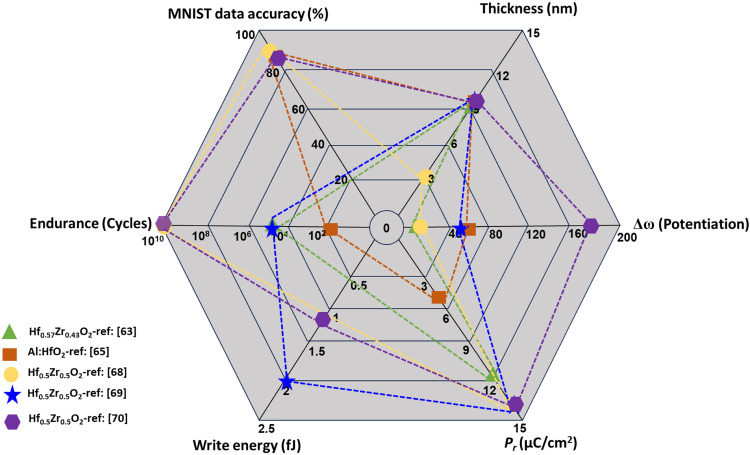
Performance of HfO_2_- and ZrO_2_-based FeFETs for different key parameters that are required for neuromorphic computing devices.

Table 2 shows some key reports on HfO_2_- and ZrO_2_-based synaptic transistors, including features like *P*_r_, endurance pulse information, write pulse duration, on/off ratio, synaptic weight, and write energy. Fig. 5 complements Table 2 in terms of the key parameters (thickness, *P*_r_, endurance, the number of conductance levels *ω*, and write energy) of HfO_2_- and ZrO_2_-based materials for application as synaptic devices. It can be concluded that reducing the thickness of the ferroelectric layer below 10 nm causes degradation of the potentiation/depression due to increased leakage currents during the program/erase process.^4^ Unfortunately, there is not much information available about the write energy and MNIST data accuracy except for Hf_0.5_Zr_0.5_O_2_ thin film FeFETs, which are considered to be key neuromorphic parameters. FeS-FET device structures are not currently being investigated on a large scale, while FeTJs and Fe memristors have been scarcely investigated.

**Table 2:** Key reports on HfO_2_- and ZrO_2_-based synaptic memory devices and their properties

Device structure	*P*_r_ (μC cm^−2^)	Endurance (cycles)	Voltage range for potentiation & depression	Write pulse duration (μs)	On/off ratio	Conductance levels Δ*ω* for potentiation/depression	Write energy (fJ)	Ref.
Al_2_O_3_/WO_*x*_/Hf_0.57_Zr_0.43_O_2_ (9.6 nm)/TiN/Si (FeFET)	∼12	10^5^	1 to 3.1 V and −0.9 to −3 V (incremental)	10	1.9	22/22	—	63
ITO/IGZO/Al:HfO_2_ (10 nm)/W/p-Si(FeFET)	∼5	10^2^	1.9 to 4 V and −1.2 to −3.3 V (incremental)	1	1 × 10^3^	70/70	—	65
W/Hf_0.57_Zr_0.5_O_2_ (5 nm)/n-SrTiO_3_/Si(FeTJ)	∼14	—	−2 V and 2 V (identical)	50	1.55	16/16	8	64
TiN/Hf_0.5_Zr_0.5_O_2_ (3.5 nm)/WO_*x*_/SiO_2_/Si(FeFET)	∼14	10^10^	−1.4 V and 2 V (unidentical)	0.02	7	25/25	—	67
TiN/Hf_0.5_Zr_0.5_O_2_(10 nm)/TiN/SiO_2_/Si (FeFET)	∼14	10^5^	0.5 V and −0.5 V (identical)	1	3 × 10^4^	50/50	2	68
TiN/Hf_0.5_Zr_0.5_O_2_ (10 nm)/WO_*x*_/SiO_2_/Si (FeFET)	∼14	10^10^	0.6 V to 5 V and −1.25 V to −5.5 V (incremental)	500	—	171/169	1.2	69
Au/Hf_0.93_Y_0.07_O_2_ (4.5 nm)/La_0.7_Sr_0.3_MnO_3_/Nb–SrTiO_3_ (Fe Memristor)	—		1.5 to 4 V and −1.5 to −4 V (incremental)	10 000	540	50/50	—	66

Just as with HfO_2_, pure or doped ZrO_2_-based thin films are promising for high-*k* gate dielectrics and are compatible with CMOS technology, and it is surprising that the material has been seldom investigated for ferroelectricity.^71–74^ In 2016, Fan *et al.* fabricated ZrO_2_ films using magnetron sputtering and stabilized the ferroelectric o-phase by substrate-induced strain to form highly textured (111) ZrO_2_ ferroelectric films.^72^ It was found that the o-phase is stabilized by applying compressive strain along the in-plane 〈112̄〉 direction. Consequently, a phase transition from the tetragonal (t-) phase to the ferroelectric o-phase occurred in the ZrO_2_ film. Lin *et al.* verified the existence of a ferroelectric o-phase in undoped ZrO_2_ films (10 nm thick) made by remote plasma ALD.^73^ More recently, Wang *et al.* reported a *P*_r_ of 8.5 μC cm^−2^ and a high relative permittivity of 39.6 in 30-nm-thick pure ZrO_2_ films grown on Si (100) *via* chemical solution deposition.^23^ Ferroelectricity in pure ZrO_2_ films is established after a wake-up operation of about 1000 cycles. According to Wang *et al.*, the wake-up process induces ferroelectricity by changing the non-ferroelectric t-phase to a ferroelectric o-phase *via* oxygen loss. In addition, it was found that an increase in film thickness (40 nm and 50 nm) weakened the ferroelectric behavior and this was ascribed to the phase transformation from the o-phase to the cubic phase as strain relaxes with thickness. In the same year, Silva *et al.* showed ferroelectricity in r-phase ZrO_2_ thin films without any wake-up process.^20^ The films were grown on (111)-oriented Nb:SrTiO_3_ (Nb:STO) using ion beam sputtering. Ferroelectricity in these ZrO_2_ thin films is attributed to the formation of a polar r-(*R*3*m*) phase under epitaxial compressive strain. Pole figure measurements of the 8-nm-thick ZrO_2_ thin films confirmed the plane distances *d*_200_*= d*_020_*= d*_002_ in the range between 2.55 Å and 2.50 Å, indicative of an r-space group. The films displayed a spontaneous polarization *P*_s_ of 20 μC cm^−2^ and a *P*_r_ of 10.8 μC cm^−2^ with an *E*_c_ of 1.5 MV cm^−1^. By converting an anti-ferroelectric t-phase to a ferroelectric o-phase, Cheema *et al.* introduced a strategy to achieve ferroelectricity in ZrO_2_ films through reduced dimensionality.^71^ The films were deposited using ALD. This work reduced the ZrO_2_ film thickness down to 5 Å and stabilized the ferroelectric o-phase. The reduced dimensionality can be achieved through hydrostatic pressure, chemical pressure, or epitaxial strain.^71^ Very recently, Silva *et al.* stabilized the o-phase in 8-nm-thick ZrO_2_ films deposited using ion beam sputtering on Nb:STO substrate.^20^ The films displayed a *P*_r_ of 9.3 μC cm^−2^ and an *E*_c_ of 1.2 MV cm^−1^ without any wake-up process. The lack of wake-up effect is attractive for next-generation memory devices. However, the potential application of ferroelectric ZrO_2_ in neuromorphics is yet to be demonstrated.

### 3.2 Doped zinc oxide films

ZnO is regarded as a conventional non-ferroelectric II–VI wurtzite semiconductor material with a band gap ∼3.4 eV.^75^ Theoretical investigations done by Moriwake *et al.*, however, reported the possibility of a spontaneous polarization *P*_s_ of ∼ 90 μC cm^−2^ in wurtzite ZnO due to the structural distortion arising from the relative displacement of cations against anions along the *c*-axis.^75,76^ This is attributed to the tensile strain experienced along the *ab*-plane along the [0001] direction. Consequently, the potential barrier obstructing the polarization rotation is reduced and the non-polar *P*6_3_/*mmc* switches between two equivalent polar states (*P*6_3_*mc*), inducing ferroelectricity.^76^

In order to make ZnO attractive as a ferroelectric tunneling resistive memory for low power non-volatile memory devices with fast switching speeds, appropriate dopants such as Li, Mn, Co, Cr, and Ti are required both to tune the band gap of ZnO and to enhance its ferroelectricity.^77–79^ Onodera *et al.* reported ferroelectric behavior in wurtzite-structured Li-doped ZnO bulk ceramics with a modest *P*_r_ of ∼ 0.044 μC cm^−2^ at ambient temperature using *P*–*E* hysteresis loop measurements. Here, the Li ion occupies the off-centered positions, replacing Zn ions. This forms electric dipoles to induce ferroelectric polarization.^79^ Yang *et al.* reported ferroelectric polarization in V-doped ZnO thin films deposited on Si(111) substrates with a *P*_r_ of ∼0.2 μC cm^−2^ using magnetron co-sputtering. The introduction of V^5+^ at Zn^2+^ sites affects the 3d–2p hybridization and induces a local dipole moment by changing the Zn(v)–O bond length.^77^ While the principal demonstration of ferroelectricity in ZnO is promising, the low *P*_r_ in these doped ZnO films obviously requires improvement, possibly by optimizing the dopant type and concentration and strain levels.^76–80^

Recently, Mg has emerged as an ideal stressor not only capable of inducing superior ferroelectric properties, but also capable of stabilizing the wurtzite structure of ZnO.^81–83^ Jacques *et al.* reported a high *P*_r_ of ∼80 μC cm^−2^ and an *E*_c_ of ∼3 MV cm^−1^ for epitaxial Zn_0.64_Mg_0.36_O (ZMO) films (thickness ∼300 nm) deposited using magnetron sputtering with pure Zn and Mg targets.^81^ To induce ferroelectric switching, an applied electric field greater than *E*_c_ had to be applied for a sufficiently long duration to wake up the polarization states. The full realization of the ferroelectric switching in ZMO films was observed after 100 cycles with the application of an electric field of ∼5 MV cm^−1^. The excess electric field of 5 MV cm^−1^ had to be applied to overcome the internal electric field in the ZMO films due to increased deep trap states attributed to the migration of defects at the interfaces.^81^ In Zn_1−*x*_Mg_*x*_O (ZnMgO) films of ∼500 nm thickness grown on single-crystal Al_2_O_3_, with *x* between 0.35 and 0.37, Ferri *et al.* reported ferroelectric switching with a *P*_r_ exceeding 100 μC cm^−2^ and an *E*_c_ less than 3 MV cm^−1^.^82^ The pronounced wake-up process in the ferroelectric ZnMgO films concluded after 100 cycles with an applied electric field greater than *E*_c_. Ferri *et al.* also demonstrated the direct integration of ZnMgO films on flexible substrates such as kapton and polystyrene. This introduces the possibility of exploring, for the first time, ferroelectric switching in wurtzite solid solutions for neuromorphic computing. Despite the promising *P*_r_ values, however, a considerable decrease of *E*_c_ is needed to make ZnO realistic for neuromorphic applications. To achieve this, we anticipate that different strategies such as epitaxial growth, doping, strain engineering, domain engineering, *etc.* can be used.

### 3.3 Scandium-doped aluminum nitride (Al_1−*x*_Sc_*x*_N) films

Pure aluminum nitride (AlN) with a wurtzite structure is generally a pyroelectric material as its polarization direction cannot be switched with an applied electric field.^69^ According to the literature, however, ferroelectricity in AlN can be induced by adding Sc to it. This is ascribed to the elongation of the Al–N bond parallel to the *c*-axis, which arises due to the formation of a metastable hexagonal phase introduced by Sc.^84–88^ The layered hexagonal phase, particularly around the Sc site, is non-polar. The increase in the Sc content flattens the ionic potential towards the hexagonal phase and therefore reduces the energy barrier, which obstructs the polarization switching in the wurtzite structure of AlN.^84–88^ As a result, Sc-doped AlN experiences ferroelectric switching.

**Fig. 6:**
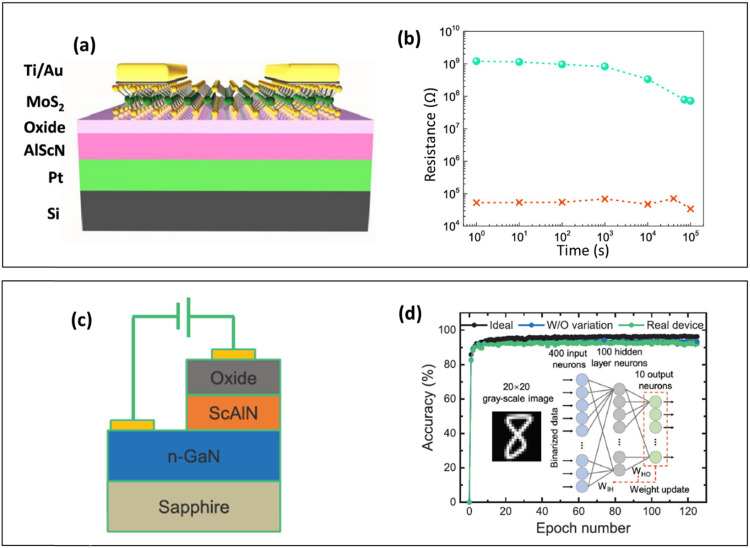
(a) Schematic diagram of a 100-nm-thick ferroelectric AlScN–MoS_2_ FeFET and (b) retention measurements made by monitoring the drain current at various time intervals of up to 100 000 s with a gate voltage of ±40 V. Copyright (2020) by American Chemical Society.^32^ (c) Illustration of an Fe memristor device using an AlScN ultra-thin film. (d) MNIST simulated classification performance of the AlScN Fe memristor showing the best recognition accuracy of 92.9% with non-linearity and cycle-to-cycle/device-to-device variations.^92^

Fichtner *et al.* reported a high *P*_r_ of ∼100 μC cm^−2^ with an *E*_c_ of ∼3 MV cm^−1^ in reactively sputtered Al_0.64_Sc_0.36_N films of thickness 400 nm deposited on oxidized Si(100) wafers.^84^ This excellent ferroelectricity in Al_0.64_Sc_0.36_N is associated with the existence of the hexagonal phase and compositional homogeneity, which allows only 180° orientation of domains. Besides, the Al_0.64_Sc_0.36_N film exhibits a piezoelectric coefficient of 15.7 pm V^−1^ with an excellent symmetry around the field axis. It should be noted that the increased Sc content above a particular limit causes a linear decrease in *E*_c_. This was concluded from the composition analysis performed by Fichtner *et al.*, where exceeding a certain threshold of Sc content impeded the ferroelectric properties again, favoring the formation of more of a non-polar layered hexagonal phase, which in turn distorts the wurtzite structure due to the expansion of the basal plane and the increasing length of the metal–nitrogen bond.^84^ Wang *et al.* fabricated Al_0.71_Sc_0.29_N thin film capacitors to demonstrate ferroelectric switching.^89^ The 100 nm Al_0.71_Sc_0.29_N thin film was deposited on Pt-coated Ti using magnetron sputtering. A wake-up operation up to 50 cycles was required to grow and stabilize the ferroelectric domains in the Al_0.71_Sc_0.29_N thin film. The Al_0.71_Sc_0.29_N thin film displayed a *P*_r_ of ∼100 μC cm^−2^ with an *E*_c_ of ∼5.5 MV cm^−1^ near room temperature. Further work performed by Wang *et al.* reported a record high *P*_r_ of ∼140 μC cm^−2^ and an *E*_c_ of ∼6.5 MV cm^−1^ in 20-nm-thick Al_0.68_Sc_0.32_N thin films near room temperature.^90^ The film was deposited on a Pt (111)/Ti/SiO_2_/Si wafer using magnetron sputtering. The formation of a strongly *c*-axis-oriented Al_0.68_Sc_0.32_N film was elucidated using X-ray diffraction and rocking curve measurements, which confirmed its highly crystalline nature with a full width at half maximum of 2.7°. The excellent ferroelectric switching in this Al_0.68_Sc_0.32_N film might be due to a combination of high crystalline nature, low film thickness, and a lower depolarization field at the surface oxide. Yasuoka *et al.* studied the effect of deposition conditions on Al_1−*x*_Sc_*x*_N thin films deposited on Pt(111)/Ti/SiO_2_/Si substrates using magnetron sputtering.^91^ The group investigated the effect of sputtering gas, film composition, and film thickness on the ferroelectric behavior of Al_1−*x*_Sc_*x*_N films. The Al_1−*x*_Sc_*x*_N films deposited under an N_2_ atmosphere showed good ferroelectricity compared with an N_2_ + Ar atmosphere. The smaller value of *P*_r_ in the Al_1−*x*_Sc_*x*_N film deposited under an N_2_ + Ar atmosphere might be attributed to the increased pinning of polarization states. The composition analysis of Al_1−*x*_Sc_*x*_N films in the range 0 < *x* < 0.34 displayed an increase in lattice parameter c up to 0.22 Å and showed a decreasing trend above this value. This indicates that the ferroelectricity is optimum for the composition *x* = 0.22 and is ascribed to the presence of more non-polar layered hexagonal phases for larger values of *x*, which contribute to the lengthening of the metal–nitrogen bond. Furthermore, the crystal structure analysis of the various thicknesses (48–140 nm) of Al_1−*x*_Sc_*x*_N films with *x* = 0.22 using rocking curve XRD analysis revealed that the preferential (002) orientation becomes predominant above 50 nm. Thus, the maximum values of *P*_r_ and the dielectric constant were found to be 129 μC cm^−2^ and 15.9, respectively, for the Al_1−*x*_Sc_*x*_N films with *x* = 0.22 and 140 nm thickness.^91^ Ryoo *et al.* reported a high *P*_r_ of ∼100 μC cm^−2^ and an *E*_c_ of >5 MV cm^−1^ in 45-nm-thick Al_0.7_Sc_0.3_N films deposited on a SiO_2_/Si substrate *via* sputtering.^27^ The enhanced ferroelectricity in the Al_0.7_Sc_0.3_N thin films arises from the imposed compressive strain, which eventually increases the *c*/*a*-lattice parameter ratio attributable to the enhanced (002)-preferred orientation. In another work, Kim *et al.* fabricated 30-nm-thick Al_0.70_Sc_0.30_N wurtzite thin films with a high *P*_r_ of 100 μC cm^−2^ with an *E*_c_ of >6 MV cm^−1^*via* sputtering deposition on a SiO_2_/Si substrate. This is ascribed to the highly *c*-axis-oriented crystal structure of the film favouring very good switching speed, eliminating the slow motion of the domain wall. Furthermore, a fatigue-free behavior (<10^5^ cycles) was observed in the film.^28^ Musavigharavi *et al.* grew 20-nm-thick ferroelectric Al_0.64_Sc_0.36_N on a SiO_2_/Si substrate by sputtering. The work reported a maximum *P*_r_ of 30 μC cm^−2^ with an *E*_c_ of 6.5 MV cm^−1^. The large polarization is attributed to the formation of a highly crystalline wurtzite structure of Al_0.64_Sc_0.36_N at lower thickness with a preferential (002) orientation achieved normal to the SiO_2_/Si substrate.^29^ Wang *et al.*, in 2023, reported ferroelectricity in ultra-thin Al_0.7_Sc_0.3_N deposited on a molybdenum (Mo) substrate using molecular beam epitaxy (MBE). The work displayed a *P*_r_ of 23 μC cm^−2^ with an *E*_c_ of > 6 MV cm^−1^.^30^ The group investigated the ferroelectric behavior of Al_0.7_Sc_0.3_N by varying the thickness from 100 to 5 nm. It is observed that the thickness decrease had a significant effect on *P*_r_ (decreases) and *E*_c_ (increases). The diminution in the ferroelectric polarization in the wurtzite Al_0.7_Sc_0.3_N ultra-thin films results from the high compressive strain induced by the scaling-down of the film thickness. Furthermore, in addition to the Janovec–Kay–Dunn (JKD) model (where *E*_c_ is proportional to *d*^−2/3^ with the film thickness *d*), the formation of a non-switchable “dead” layer or dielectric interfacial layer, or a finite screening length of the electrodes, contributes to the increase in *E*_c_ with the scaling down of the thickness. Zheng *et al.* developed ultra-thin Al_0.72_Sc_0.28_N films (thickness ∼5 nm) deposited on a sapphire substrate using physical vapor deposition and investigated their ferroelectric properties. The work showed *P*_r_ > 100 μC cm^−2^ with *E*_c_ ∼9.2 MV cm^−1^.^31^ This work suggests that the high *E*_c_ value for lower thickness may be due to the decrease in the *c*/*a* ratio arising from the Sc alloying ratio or from the film stress. Liu *et al.* fabricated an FeFET device, as shown in Fig. 6(a), composed of 100-nm-thick sputtered Al_0.71_Sc_0.29_N integrated with a two-dimensional (2D) molybdenum disulfide (MoS_2_) channel.^32^ The device achieved a high *P*_r_ of 80 μC cm^−2^ and an *E*_c_ of 6 MV cm^−1^. Furthermore, the device exhibited an excellent on/off ratio of ∼10^6^, an endurance of 10^4^ cycles, and a good retention of 10^5^ s, which was however measured at a gate voltage of ±40 V, which is a very high writing voltage (Fig. 6(b)). The 2D MoS_2_ channel minimizes the depolarization field by compensating the incomplete charge induced by the ferroelectric Al_0.71_Sc_0.29_N layer, consequently providing prolonged retention and minimal read-disturb. Very recently, Wang *et al.* studied the ferroelectric and analog resistive switching of memristors in 5 nm wurtzite Al_0.7_Sc_0.3_N with gallium nitride (GaN) as the semiconductor channel grown on a sapphire substrate using MBE, as shown in Fig. 6(c).^92^ The device demonstrated its potential as a candidate for nonlinear ferroelectric resistive memory arrays with a high on/off ratio of 10^5^, an endurance of >10^4^ cycles, and a good retention of 10^5^ s. Furthermore, the work displayed eight conductance states during potentiation and depression for the incremental pulse scheme with voltages ranging from −7.6 V to −8.24 V and 1.9 V to 3.564 V, respectively. However, some reports suggest that a minimum of ten conductance states during potentiation/depression are required for the effective training process by an ANN in the analog domain.^92^ Nevertheless, the device yielded a recognition accuracy of 92.9% from MNIST simulation (Fig. 6(d)).

Based on these promising demonstrations, we foresee that more studies on these high-*P*_r_ Al_1−*x*_Sc_*x*_N films could produce further synaptic elements with multi-level polarization states essential for analog conduction modulation during potentiation/depression with CMOS compatibility. Strategies like domain and strain engineering, as well as thickness reduction, could potentially be explored for reducing *E*_c_ to enhance the potential neuromorphic applicability.

### 3.4 Layered bismuth oxide films

Bismuth oxide is a classical ferroelectric material with a very high Curie temperature (∼673 K) and excellent cycling endurance (∼10^12^ cycles).^93,94^ This discovery builds on the finding of in-plane ferroelectricity in BiO layers in complex layered bismuth oxide compositions, *e.g.*, Bi_2_AlMnO_6_ and Bi_2_NiMnO_6_. In these materials, the growth of the layered structure occurs more in the horizontal than in the vertical direction. It is reported that out-of-plane ferroelectric properties offer higher polarization switching than the in-plane orientation due to more uniform grains and ordered domains.^95–98^ Very recently, Yang *et al*. reported strong out-of-plane ferroelectricity in 1-nm-thin Sm-substituted bismuth oxide (Bi_1.8_Sm_0.2_O_3_) grown on an Al_2_O_3_ substrate using sol–gel spin-coating.^99^ The 1-nm-thin Bi_1.8_Sm_0.2_O_3_ (BSO) film exhibited a *P*_r_ of 17 μC cm^−2^, which is high compared with other ultra-thin films such as HZO, CuInP_2_S_6_, BaTiO_3_, BiFeO_3_, and PbZr_0.2_Ti_0.8_O_3_. Here, the Bi layers at the interface serve as strain bearers to relax the lattice mismatch from the substrate, thus maintaining an unstrained growth of the film. Even though the reduced thickness affects the ferroelectricity in the BSO film due to surface charges and leakage, the remarkable *P*_r_ arises due to the incorporation of the Sm dopants, which preserves the ferroelectric phase in the BSO and maintains a high polarization by suppressing the depolarization field. Furthermore, Sm promotes the tolerance of the layered Bi structure to uphold the stability of the ferroelectric structure at low dimensions. A slightly thicker film of 4.56-nm-thin BSO displayed a high *P*_r_ of 50 μC cm^−2^ with an *E*_c_ of about 0.75 MV cm^−1^. In addition, the film exhibited an excellent cycling endurance (∼10^8^ cycles) and outstanding thermal stability (up to 493 K). The demonstrated *E*_c_ and *P*_r_ values at highly scaled film thicknesses are promising for neuromorphic applications such as non-destructive read/write operations and synaptic functionality. Hence, this new material warrants further deeper explorative studies of its neuromorphic behavior.

**Fig. 7:**
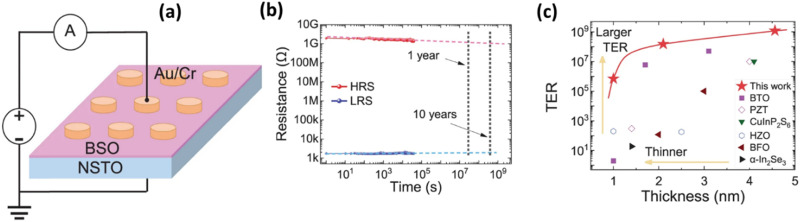
(a) Schematic diagram of a 4.6-nm-thin ferroelectric Bi_1.8_Sm_0.2_O_3_ (BSO) FeTJ device structure and (b) retention measurement indicating prolonged retention for more than 10 years by extrapolation. (c) Comparison of tunnelling electroresistance (TER) values of the BSO FTJ device with other ferroelectric materials and thicknesses.^34^

Very recently, Gia *et al.* demonstrated an FeTJ based on an ultra-thin BSO film of thickness 4.6 nm deposited on Nb:STO using a chemical solution method (Fig. 7(a)).^34^ The FeTJ exhibited long retention time (10 years, Fig. 7(b)) and exceptional cycling endurance (10^9^). The stable tetragonal-like phase induced by Sm substitution provided prolonged retention in the BSO-based FeTJs by reducing the depolarization field. Furthermore, the BSO film showed an excellent *P*_r_ of 50 μC cm^−2^ and a very low *E*_c_ of 0.75 MV cm^−1^. Moreover, the FeTJ displayed a large tunnelling electroresistance compared to other materials attributable to the large polarization maintained at the minute thickness (Fig. 7(c)). Consequently, both the ferroelectric barrier height and barrier width at the depletion region surface of Nb:STO were modulated. In this work, a single BSO-based FeTJ demonstrated 32 conductance states without any write-termination process. Hence, this new material warrants further deeper explorative studies of its neuromorphic behavior.

A summary comparing *P*_r_, *E*_c_, endurance, retention, and write energy of the different materials, together with neuromorphic performance parameters, is shown in Table 3. It is possible to conclude that Hf_0.5_Zr_0.5_O_2_ partially meets the requirements of neuromorphic applications. In the case of Hf_0.5_Zr_0.5_O_2_, we took the best performance^56^ from Fig. 5 to compare the neuromorphic parameters with other materials. However, these novel ferroelectrics are in the early stage of development and strong performance improvements are eagerly anticipated.

**Table 3:** Important performance parameters for achieving neuromorphic functionality for the different materials. For each material, we have selected the best-performing single device based on *P*_r_ and *E*_c_ values, and the other entries in each column are for the same device. Illustrative figures for each parameter are taken from ref. 20, 25, 34, 69, and 92. Here, the tick sign indicates that the ferroelectric material meets each particular parameter requirement for neuromorphics, the cross sign means the performance has not been reached and a dash means that the parameters have not yet been explored

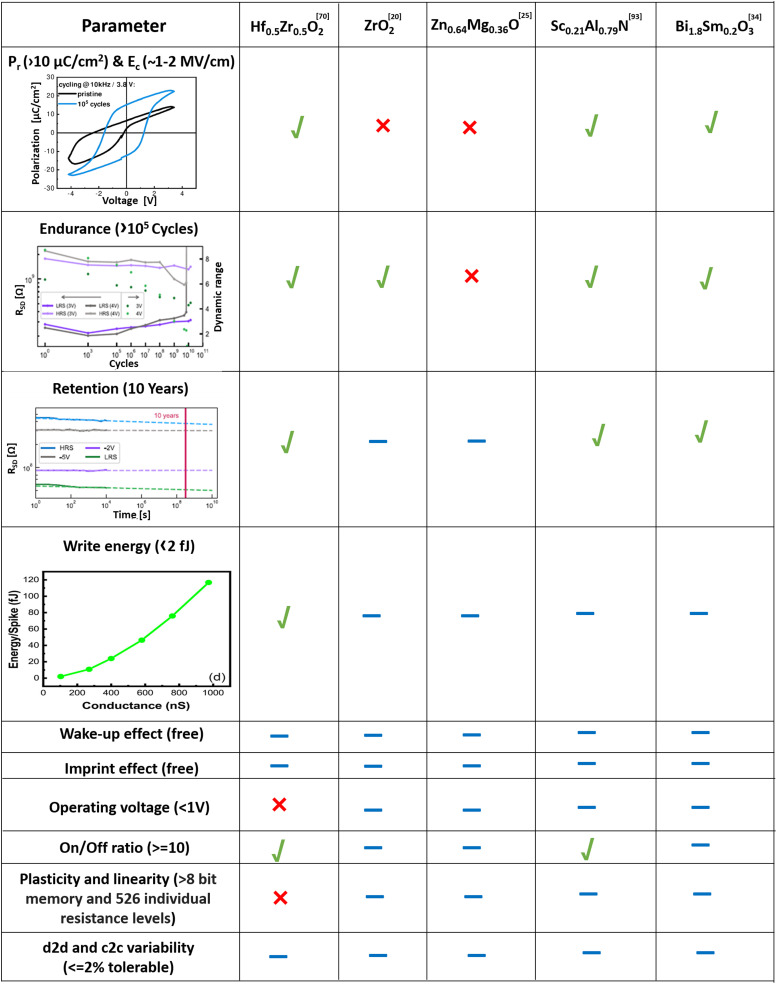

## 4. Concluding remarks

The recent discoveries of ferroelectricity in binary and pseudo-binary nanoscale oxide thin films offer great possibilities for miniaturized non-volatile memory and neuromorphic computing devices. We have reviewed the most promising of these oxide thin film materials, and examples are summarized in Table 1. These new materials show high polarization in the nano-regime, which is particularly important for ultra-scaled devices. Most importantly, HfO_2_- and ZrO_2_-based non-volatile FeFETs have already been established as promising contenders for synaptic elements in neuromorphic devices due to their established deposition methods, high ferroelectric polarization, scalability, high endurance, defect-free operation mechanism, and CMOS compatibility.^63–68^ The impressive performance of HfO_2_- and ZrO_2_-based thin film FeFETs is getting significant attention due to high scalability, good endurance, and CMOS compatibility.^4^

In addition, these group-IV-based oxide materials exhibit important performance metrics, including good linearity/symmetry, multilevel states, high on/off ratios and low write energy characteristics, which are desirable for neuromorphic applications.^4,63–65,67–69^ However, HfO_2_- and ZrO_2_-based thin films typically struggle with parameters such as imprint, wake-up, fatigue, and consequently with uniform cycling endurance. Also, reducing the film thickness, as is required in some cases, may increase *E*_c_ and therefore necessitate higher operating voltages, leading to higher leakage currents during program/erase operations.

Based on the initial demonstrations of ferroelectricity in Sc-AlN, ZnMgO, and BiSmO_3_, there is potential for these materials for neuromorphic computing, too. However, while ZnMgO exhibits exceptional *P*_r_ values comparable to HfO_2_ and ZrO_2_ thin films, as of yet the large coercive field and thickness of ZnMgO present challenges for its integration as a neuromorphic computing device. Also, Sc-doped AlN and ZnMgO suffer from problems of low endurance and the requirement for wake-up cycling. Furthermore, parameters like on/off ratio, potentiation/depression, write energy, synaptic weight, *etc.* remain unexplored in ferroelectric ZrO_2_ and ZnMgO. Also, while very recently studied Bi_1−*x*_Sm_*x*_O_3_^34^ meets some performance parameter requirements for a synaptic element, all these performance parameters need to be explored in a substrate compatible with CMOS fabrication processes.

For all the aforementioned oxide materials, strategies should be implemented to control the ferroelectric phase stability and reduce oxygen vacancy concentrations. More studies on epitaxial films are useful in this regard as they serve as model systems to tune the structure carefully without influences of grain boundaries and mixed phases.

With a good understanding of the fundamental properties of materials and their control, domains can then be engineered to achieve low-voltage multi-level synaptic plasticity with low variability for neuromorphic applications. For example, the controlled design of sufficiently many domains in a thin film would allow a more fine-grained tuning of multiple polarization levels for quasi-analog synaptic plasticity and at the same time would improve variability due to the suppression of stochastic switching of only a few domains. Finally, we note that there are also many opportunities to expand the aforementioned ferroelectric thin film systems for applications beyond memory and neuromorphic computing, namely in pyroelectric- and piezoelectric-based energy harvesters, transducers, energy storage, *etc.*^100–105^

## Conflicts of interest

There are no conflicts to declare.

## Supplementary Material
